# Trichostatin A, a histone deacetylase inhibitor suppresses NADPH Oxidase 4‐Derived Redox Signalling and Angiogenesis

**DOI:** 10.1111/jcmm.12885

**Published:** 2016-06-14

**Authors:** Nora Y. Hakami, Gregory J. Dusting, Hitesh M. Peshavariya

**Affiliations:** ^1^ Centre for Eye Research AustraliaRoyal Victorian Eye and Ear HospitalEast MelbourneVICAustralia; ^2^ OphthalmologyUniversity of MelbourneDepartment of SurgeryEast MelbourneVICAustralia; ^3^ Department of Pharmacology and TherapeuticsUniversity of MelbourneMelbourneVICAustralia; ^4^ Faculty of Applied Medical SciencesKing Abdulaziz UniversityJeddahSaudi Arabia

**Keywords:** Nox4, histone acetyltransferases, histone deacetylase inhibitor, trichostatin A, TGFβ1

## Abstract

Histone deacetylase (HDAC) inhibitors are known to suppress abnormal development of blood vessels. Angiogenic activity in endothelial cells depends upon NADPH oxidase 4 (Nox4)‐dependent redox signalling. We set out to study whether the HDAC inhibitor trichostatin A (TSA) affects Nox4 expression and angiogenesis. Nox4 expression was measured by real time PCR and Western blot analysis in endothelial cells. Hydrogen peroxide (H_2_O_2_) was measured by amplex^®^ red assay in endothelial cells. Nox4 was knocked down by Nox4 shRNA. *In vitro* angiogenic activities such migration and tubulogenesis were assessed using wound healing and Matrigel assays, respectively. *In vivo* angiogenic activity was assessed using subcutaneous sponge assay in C57Bl/6 and Nox4‐deficient mice. Trichostatin A reduced Nox4 expression in a time‐ and concentration‐dependent manner. Both TSA and Nox4 silencing decreased Nox4 protein and H_2_O_2_. Mechanistically, TSA reduced expression of Nox4 *via* ubiquitination of p300‐ histone acetyltransferase (p300‐HAT). Thus, blocking of the ubiquitination pathway using an inhibitor of ubiquitin‐activating enzyme E1 (PYR‐41) prevented TSA inhibition of Nox4 expression. Trichostatin A also reduced migration and tube formation, and these effects were not observed in Nox4‐deficient endothelial cells. Finally, transforming growth factor beta1 (TGFβ1) enhanced angiogenesis in sponge model in C57BL/6 mice. This response to TGFβ1 was substantially reduced in Nox4‐deficient mice. Similarly intraperitoneal infusion of TSA (1 mg/kg) also suppressed TGFβ1‐induced angiogenesis in C57BL/6 mice. Trichostatin A reduces Nox4 expression and angiogenesis *via* inhibition of the p300‐HAT‐dependent pathway. This mechanism might be exploited to prevent aberrant angiogenesis in diabetic retinopathy, complicated vascular tumours and malformations.

## Introduction

The NADPH oxidase (Nox) family of enzymes is the major source of reactive oxygen species (ROS) in the vascular diseases 1. The enzyme is comprised of seven isoforms (Nox1 to Nox5, Duox1 and 2) amongst which Nox1, Nox2, Nox4 and Nox5 are expressed by endothelial cells 2, 3. These isoforms differ in terms of their sub‐cellular localization, requirement for their cytosolic subunits, the type of ROS they produce and tissue specific expression 2. Unlike other Nox family members, Nox4 is constitutively active and normally produces hydrogen peroxide (H_2_O_2_) rather than superoxide 4. Several physiological and pathological stimuli such as, transforming growth factor beta 1 (TGFβ1) 5, 6, prostacyclin 7, tumour necrosis factor alpha 8, hypoxia 9, 10 and cell confluence 11 can up‐regulate Nox4 expression in endothelial cells. Nox4 expression or activity is thought to be involved in many endothelial cell functions including regulation of proliferation, migration and angiogenesis both *in vitro* and *in vivo* 5, 7, 8, 9, 12, 13, 14.

Acetylation of chromosomal histones by histone acetyltransferases (HATs) opens the chromatin and allows binding of transcription factors to the promoter sites leading to enhanced gene expression. In addition, HATs act as co‐activators and directly regulate activity of non‐histone transcription factors that, in turn, regulate gene expression 15, 16. For instance, shear stress induces the acetylation of p65, a subunit of nuclear factor κB (NF‐κB) transcription factor, which in turn results in transcriptional activation of endothelial nitric oxide synthase (eNOS) expression by the p300‐histone acetyltransferase (p300‐HAT)‐dependent pathway 17. Conversely histone deacetylases (HDACs), a class of enzymes that remove the acetylation signature from histone and non‐histone proteins, counteract HATs. This interplay between HATs and HDACs is a fundamental process that regulates the acetylation status of transcription factors and fine tunes gene expression.

Thus, HATs and HDACs have opposite actions in terms of gene regulation. Recently, we found that inhibition of p300‐HAT using either a pharmacological inhibitor garcinol or p300 siRNA, reduced Nox4 expression in endothelial cells 7. Similarly, we in the present study show that inhibition of HDAC surprisingly also reduces Nox4 expression in endothelial cells. Since HATs and HDACs generally have opposite actions in terms of gene regulation, but it is unclear how inhibition of both enzymes reduced expression of Nox4. Therefore, in the present study, we have delineated how inhibition of HDACs interferes with p300‐HAT activity and Nox4 expression in endothelial cells.

Histone deacetylase inhibitors have been of considerable interest for many diseases including treatment of cancer 18, slowing cardiac hypertrophy and heart failure 19 as well as suppressing inflammation and immune diseases 20, 21. Histone deacetylase inhibitors have also been shown to reduce angiogenesis in the eye and tumour 18, 22, 23. The molecular mechanisms underlying the anti‐angiogenic effect of HDAC inhibitors have been attributed to down‐regulation of hypoxia‐inducible factor‐1α (HIF‐1α) and VEGF expression under hypoxic conditions 22. Histone deacetylase inhibitors have also been shown to reduce angiogenesis under non‐hypoxic conditions through alternative mechanisms such as destabilization of eNOS mRNA and reduction of nitric oxide production 24. We and others have previously shown that Nox4 plays an important role in angiogenesis 5, 9, 14, 25 and it regulates the activity of both HIF‐1α 14 and eNOS 5, 9. Therefore, we also investigated the requirement of Nox4 signalling for the effects of HDAC inhibitor TSA on angiogenesis *in vitro* and *in vivo*.

## Materials and methods

### Cell culture

A human umbilical vein endothelial cells (HUVECs) were purchased from Lonza, Victoria, Australia, and human microvascular endothelial cells (HMECs) were kind gifts from Centre for Disease Control and Prevention, Atlanta, USA. Mouse lung endothelial cells (MLECs) were immortalized using SV‐40T antigen. All endothelial cells types were cultured in EGM‐2 Bullet Kit with 5% foetal bovine serum (FBS), known as EGM‐2 growth media (Lonza, Mount Waverley, VIC, Australia). Human dermal fibroblasts were cultured in DMEM with 10% FBS. Endothelial and fibroblast cells were cultured in standard cell culture conditions using a 5% CO_2_ incubator at 37°C. All high quality chemicals were purchased from Sigma‐Aldrich, Castle Hill, NSW, Australia unless otherwise specified.

### Experimental setup

Unless otherwise specified, the cells were treated with HDAC inhibitors trichostatin A (TSA; 0.33 μM = 100 ng/ml), sodium butyrate (NaB), valproic acid (VPA) or TGFβ1 (10 ng/ml) for 6 hrs before cell harvest. In some cases, the cells were pre‐treated with p300‐HAT inhibitor curcumin (50 μM; Sigma‐Aldrich) or ubiquitin‐activating enzyme inhibitor PYR‐41 (2.5 μM; Merck Millipore, Billerica, MA, USA) for 1 hr before addition of TGFβ1 (10 ng/ml) or TSA (0.33 μM).

### Adenovirus infection

We silenced Nox4 gene expression using adenoviral vectors expressing small interfering RNA targeting human Nox4 nucleotides 418–436 from the start codon (Adv‐Nox4i) as described previously 5. Adenovirus expressing green fluorescent protein (Adv‐GFP) was used as a control. Cells were infected with 500 MOI (HUVECs) or 200 MOI (HMECs) of Adv‐GFP or Adv‐Nox4i for 24 hrs in Opti‐MEM medium (Life Technologies, Tullamarine, VIC, Australia) and allowed to recover in EGM‐2 growth medium for another 24 hrs. All experiments were performed 48 hrs after infection.

### siRNA Transfection

Transfections were performed using DharmaFECT‐1 siRNA reagents (Dharmacon, Lafayette, CO, USA) following the manufacturer's recommendations. Typically, HMECs 10^5^/well were plated in a 6‐well plate 1 day prior to transfection. On the second day, the cells were treated with 100 nM of either control siRNA or p300‐SMART PULL in Opti‐MEM medium and DharmaFECT‐1 for 6 hrs. Transfected cells were washed with PBS and replaced with EBM‐2 growth medium. After 48 hrs, cells were deprived with serum free medium and treated with TGFβ1 (10 ng/ml) for 6 hrs. The cells were collected for gene expression analysis.

### Gene expression analysis

Cells (10^5^ cells/well) were seeded in 6‐well plates. Serum deprived cells were treated with various inhibitors or TGFβ1. Total RNA from treated cells was extracted with the TRI‐reagent according to manufacturer's instructions (Ambion, Austin, TX, USA) and reverse‐transcribed to cDNA using TaqMan high performance reverse transcription reagents (Applied Biosystems, Life Technologies) at 25°C for 10 min., 37°C for 2 hrs followed by 85°C for 5 sec. in a Thermal cycler (BioRad‐DNA Engine; Bio‐Rad, Gladesville, NSW, Australia). The quantitative real‐time PCR reactions were performed in a 7300 system (Applied Biosystems, Life Technologies) by using TaqMan Universal PCR master mix and pre‐designed gene specific probes and primer sets for Nox2 (Hs00166163_m1), Nox4 (Hs01558199_m1 and Mm00479246_m1), Nox1 (Hs002455589_m1), Nox5 (Hs00225846_m1), EP300 (Hs00914223_m1) and NOS3 (Hs00167166_m1). The cycle threshold (CT) values form all quantitative real‐time PCR experiments were analysed using ^ΔΔ^CT method. Data were normalized to glyceraldehyde 3‐phosphate dehydrogenase (GAPDH; human 4326317E and mouse 4352339E) and expressed as fold changes over that in control treatment group.

### Amplex^®^ red assay

Extracellular H_2_O_2_ levels were detected using amplex^®^ Red assay kit (Molecular Probes, Life Technologies) according to manufacturer's instructions. HMECs or MLECs (10^5^ cells/well) were seeded in a 6‐well plate. Serum‐deprived cells were treated with and without TGFβ1 (10 ng/ml) or TSA for 6 hrs. Following treatments, trypsinized cells were suspended in Krebs‐HEPES buffer (HBSS, in mM: NaCl 98.0, KCl 4.7, NaHCO_3_ 25.0, MgSO_4_ 1.2, 137 KH_2_PO_4_ 1.2, CaCl_2_ 2.5, d‐glucose 11.1 and Hepes‐Na 20.0) containing Amplex^®^ Red reagent (10 μM) and horseradish peroxidase (0.1 U/ml). Fluorescence was then measured with excitation and emission at 550 and 590 nm, respectively, using a polarstar microplate reader (BMG Labtech, Ortenberg, Germany) at 37°C. Fluorescence values were normalized to cell numbers determined by Alamar^®^ Blue cell viability assays as according to manufacturer's instructions (Life Technologies).

### Western blot analysis

Cells (2.5 × 10^5^ cells/well) were cultured in 6‐well plates, and protein was extracted as previously described 7. Equal amounts of protein were then separated by electrophoresis using gradient SDS‐PAGE gel in case of Nox4 and 6% SDS‐PAGE gels of p300, and transferred to nitrocellulose membranes (Amersham, GE Healthcare, Parramatta, NSW, Australia). After blocking with 5% non‐fat milk in a buffer containing Tris‐HCl (20 mM, pH 7.5), NaCl (100 mM) and Tween‐20 (0.1%), respective membranes were incubated at 4°C overnight with either primary antibodies against Nox4 (rabbit monoclonal anti Nox4, 1:1000; Abcam, Melbourne Australia), primary rabbit polyclonal anti p300 (C‐20; 1:200; Santa Cruz Biotechnology, Dallas, TX, USA), and mouse monoclonal anti GAPDH (1:1000; Merck Millipore) antibodies were used. Proteins were detected using an enhanced chemiluminescence detection kit (GE Healthcare, Parramatta, NSW, Australia) with horseradish peroxidase conjugated to appropriate secondary antibodies (Bio‐Rad). The image was captured and processed using CanoScan 8800F/PhotoStudio 5.5 software (Melville, NY, USA).

### P300‐HAT immunoprecipitation

Human microvascular endothelial cells (2 × 10^6^) were washed once in ice‐cold PBS before scraping them off at 4°C with lysis buffer containing protease inhibitor. The sample protein concentration was adjusted to 1 mg/ml with lysis buffer and incubated with 10 μg of polyclonal anti‐p300‐HAT antibody (C‐20; Santa Cruz Biotechnology) overnight. Protein‐G agarose (Sigma‐Aldrich) bead suspension was added to each sample to isolate the p300‐HAT antigen‐antibody complex. Samples were spun down for 1 min. at 14,000 *g* and washed three times with cold PBS. p300‐HAT antigen antibody complex was resuspended in 2× loading buffer [125 mM Tris HCl, pH 6.8, 4% (w/v) SDS and 10% (v/v) Glycerol, 1% (v/v) 2‐mercaptoethanol] and heated at 95°C for 5 min. P300 protein and ubiquitnation of p300 were detected using polyclonal anti‐p300 antibody (C‐20, 1:200) and mouse monoclonal anti‐ubiquitin antibody (1:1000; Boston Biochem, Cambridge, MA, USA) respectively.

### Migration

The wound healing assay is an *in vitro* model to explore effects on endothelial cell proliferation and migration during closure of a cellular monolayer wound. HUVECs (1 × 10^5^ cells/well) or HMECs (1.5 × 10^5^) or MLECs (1 × 10^5^) were seeded in 12‐well plates. After 24 hrs, two perpendicular wounds were created using 1 ml pipette tips. Cells were washed three times with PBS and treated with EGM‐2 growth media containing 2% FBS in the absence and presence of TSA (0.33 μM) for 16 hrs at 37°C, 5% CO_2_. Images were captured under 10× magnification at time 0 and 16 hrs. Three different areas of the wound were measured using Image J software. Values were then expressed as the percentage wound closure at time zero and at 16 hrs.

### Tube formation assay

Serum‐deprived cells (1.5 × 10^4^ cells/well) were seeded on growth factor reduced Matrigel (50 μl) in 96 well plate and treated with or without TSA (0.33 μM). After 8 hrs, images were taken under 10× magnifications using an Olympus inverted light/fluorescent microscope (Model No. IX81). Tube length was measured using Image J software (National institute of Health, Bethesda, MD, USA) from 10 random fields and normalized to controls.

### *In vivo* angiogenesis

Animal study has been conducted in accordance with St. Vincent's Hospital Animal Ethics Committee guidelines (Melbourne, Victoria, Australia) and the Australian National Health and Medical Research Council guidelines for the care and health of animals (AEC 006‐13). The subcutaneous sponge model was used to determine the effects of TGFβ1 on angiogenesis *in vivo* as described previously 5. UV sterilized polyvinyl alcohol (PVA) sponge discs (8 mm diameter × 2 mm thickness from PVA Unlimited, Warsaw, IN, USA) were soaked in either saline (120 μl/sponge) or TGFβ1 solution (10 ng/ml; 120 μl/sponge) and implanted under the dorsal skin of 10 weeks old male C57BL/6 wild type (WT) mice, Nox4 knockout (Nox4 KO) mice 26 (kindly provided by Prof Karl‐Heinz Krause, University of Geneva) using general anaesthetic agents ketamine (100 mg/kg) and xylazine (IP 10 mg/kg) intraperitoneally. A similar experiment was performed to test the effect of TSA on TGFβ1 induced‐angiogenesis in male C57BL/6 of the same age. After implanted of saline and TGFβ1‐soaked sponge discs, mice were treated with either vehicle (1% DMSO in saline) or TSA (1 mg/kg) intraperitoneally every 48 hrs for 14 days. In both experiments, mice were killed using lethabarb (IP 200 mg/kg) after 14 days and sponges harvested and cleaned of connective tissues. Haemogloboin content assay indicates formation of new perfused vessels. In this assay, sponges were incubated with 500 μl of red blood cells lysis buffer (in mM; NH_4_Cl 200, NaHCO_3_ 20, ethylenediaminetetraacetic acid 1) for 1 hr at 37°C. The supernatant was collected by centrifugation at 5000 × g for 10 min. The concentration of haemoglobin in the supernatant was determined at an absorbance of 550 nm and compared with a standard curve of purified bovine haemoglobin using a haemoglobin assay kit (Drabkin's reagent).

### Data and statistics

Values are mean ± S.E.M., all the experiments were carried out in replicates using at least four independent cell cultures. Comparisons between two and more treatments were performed by means of Student's unpaired *t*‐test and anova, respectively. If the anova demonstrated a significant interaction between variables, post‐hoc Tukey analysis was performed for multiple‐comparison. A value of *P* < 0.05 was regarded as statistically significant.

## Results

### HDAC inhibitors reduce Nox4 expression and H_2_O_2_ formation

**Figure 1:**
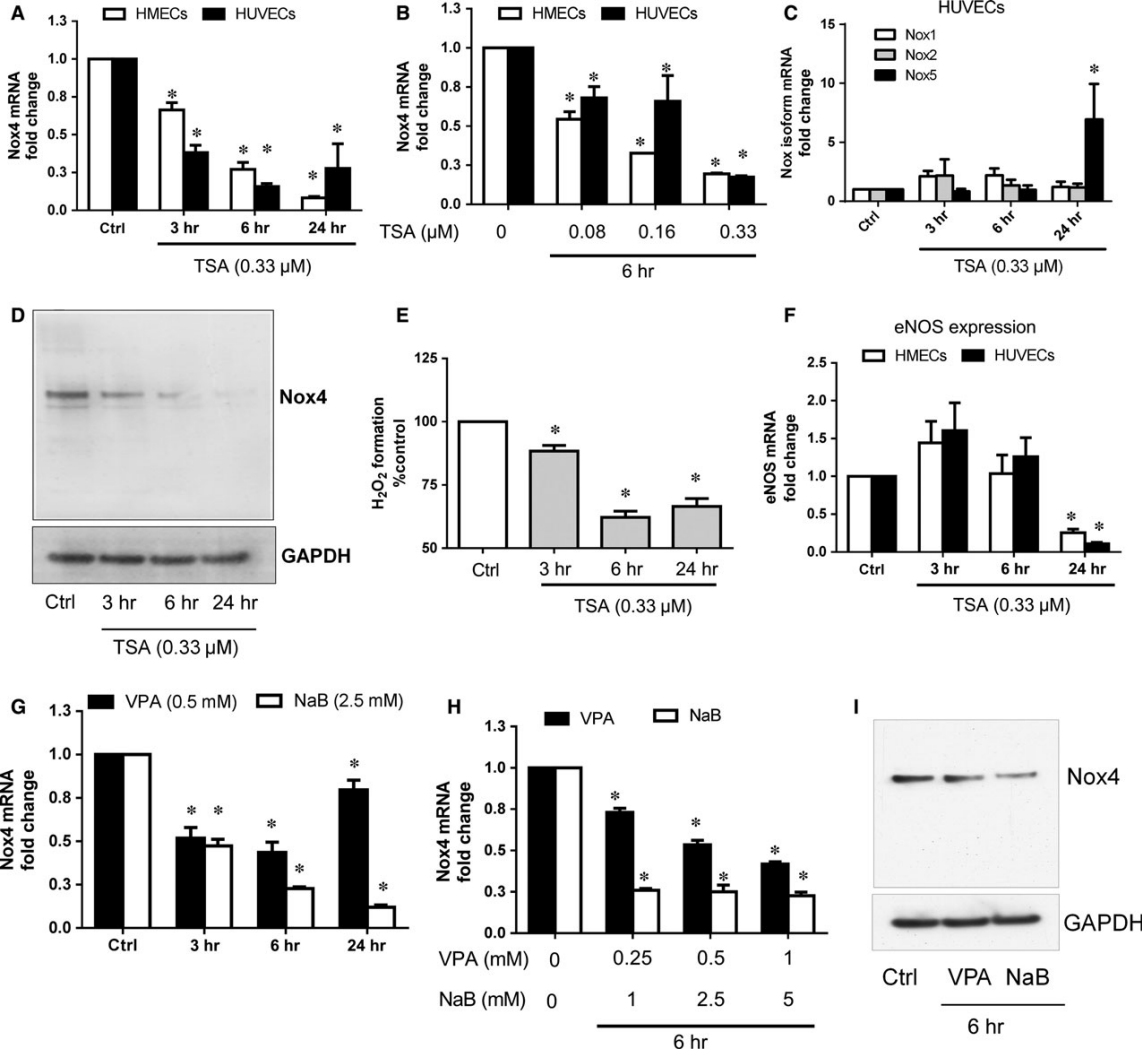
Effect of TSA on Nox4 expression and H_2_O_2_ formation in endothelial cells. Trichostatin A (TSA; 0.33 μM = 100 ng/ml) reduced Nox4 mRNA levels in a time‐ (**A**; 3–24 hrs) and concentration‐dependent (**B**; 0.08–0.33 μM) manner in HMECs (open) and HUVECs (closed). (**C**) TSA (0.33 μM) does not affect Nox1 and Nox2 mRNA expression at any time point whereas increased Nox5 mRNA expression at 24 hrs time point in HUVECs. (**D**) TSA (0.33 μM) decreased Nox4 protein levels compared to control in HMECs as shown in a representative Western blot. (**E**) H_2_O_2_ generation as detected by Amplex Red assay was reduced following 3, 6 and 24 hrs treatment of TSA (0.33 μM) in HMECs. (**F**) TSA (0.33 μM) reduced eNOS mRNA levels only at 24 hrs time point in HMECs (open) and HUVECs (closed). Other HDAC inhibitors such as valproic acid (VPA) and sodium butyrate (NaB) also reduced Nox4 mRNA levels in a time‐ (**G**; 3–24 hrs) and concentration‐dependent (**H**; VPA 0.25–1 mM and NaB 1–5 mM) manner in HMECs. (**I**) VPA (0.5 mM) and NaB (2.5 mM) diminished Nox4 protein levels compared to control in HMECs as shown in a representative Western blot. All data are mean ± S.E.M. from four independent experiments, *P < 0.05 from control without treatment.

To study the involvement of HDACs on Nox4 gene regulation, HDAC inhibitors such as TSA, VPA and NaB were used. TSA (0.33 μM) decreased Nox4 gene expression in a time‐ (Fig. 1A) and concentration‐dependent manner (Fig. 1B) in both HMECs and HUVECs. Expression of Nox1 and Nox2 did not change after 3–24 hrs treatments with TSA, whereas Nox5 expression markedly increased at 24 hrs in HUVECs (Fig. 1C). Reduction of Nox4 mRNA considerably reduced Nox4 protein level (Fig. 1D) and H_2_O_2_ production (Fig. 1E) after TSA treatment from 3 to 24 hrs in HMECs. TSA also decreased eNOS gene expression only at 24 hrs time point in both HMECs and HUVECs (Fig. 1F). Similarly, other classes of HDAC inhibitors VPA and NaB also blocked Nox4 gene expression in a time‐ (Fig. 1G) and concentration‐ dependent manner (Fig. 1H) in both HMECs and HUVECs. Reduction of Nox4 gene expression was also confirmed at protein level and both VPA (0.5 mM) and NaB (2.5 mM) reduced Nox4 protein at 6 hrs (Fig. 1I) in HMECs. These findings indicate that HDAC inhibitors reduce basal level of Nox4 expression and H_2_O_2_ formation in endothelial cells.

**Figure 2:**
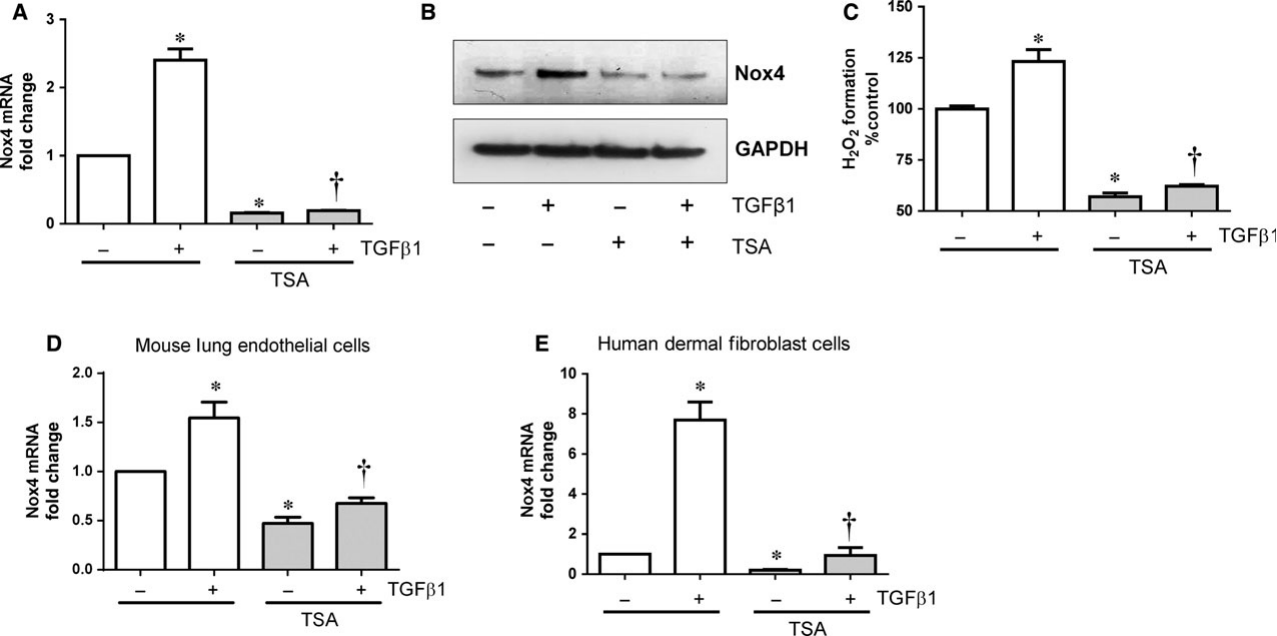
Effect of TSA on TGFβ1‐induced Nox4 expression and H_2_O_2_ formation in endothelial cells. Pre‐treatment (1 hr) of HMECs with TSA (0.33 μM) suppressed TGFβ1 (10 ng/ml‐6 hrs)‐induced (**A**) Nox4 gene expression and (**B**) Nox4 protein expression as shown in a representative Western blot. (**C**) Basal and TGFβ1 (10 ng/ml)‐induced H_2_O_2_ generation was abolished in HMECs pre‐treated with TSA. Similarly pre‐treatment of (**D**) mouse lung endothelial cells and (**E**) human dermal fibroblast cells with TSA (0.33 μM) also suppressed basal and TGFβ1 (10 ng/ml‐6 hrs)‐induced Nox4 gene expression. All data are mean ± S.E.M. from 4 to 6 independent experiments, *P < 0.05 from control without treatment; †*P* < 0.05 from treated cells with TGFβ1.

Next, we tested the effect of TSA on TGFβ1‐induced Nox4 expression and activity. TGFβ1 induced Nox4 mRNA (Fig. 2A), protein (Fig. 2B) and H_2_O_2_ formation (Fig. 2C) which were all blocked by TSA (Fig. 2A–C) in HMECs. We also confirmed the effect of TSA on Nox4 expression in MLECs and human primary dermal fibroblasts. TGFβ1‐induced Nox4 expression was markedly reduced by TSA in MLECs derived from WT mice (Fig. 2D). Similarly, TSA reduced both basal and TGFβ1‐induced Nox4 expression in human dermal fibroblasts (Fig. 2E). These results indicate that HDAC inhibitor TSA reduced basal as well as TGFβ1‐induced Nox4 expression in endothelial and fibroblast cells.

### Trichostatin A induces ubiqutination and proteasome degradation of p300‐HAT

**Figure 3:**
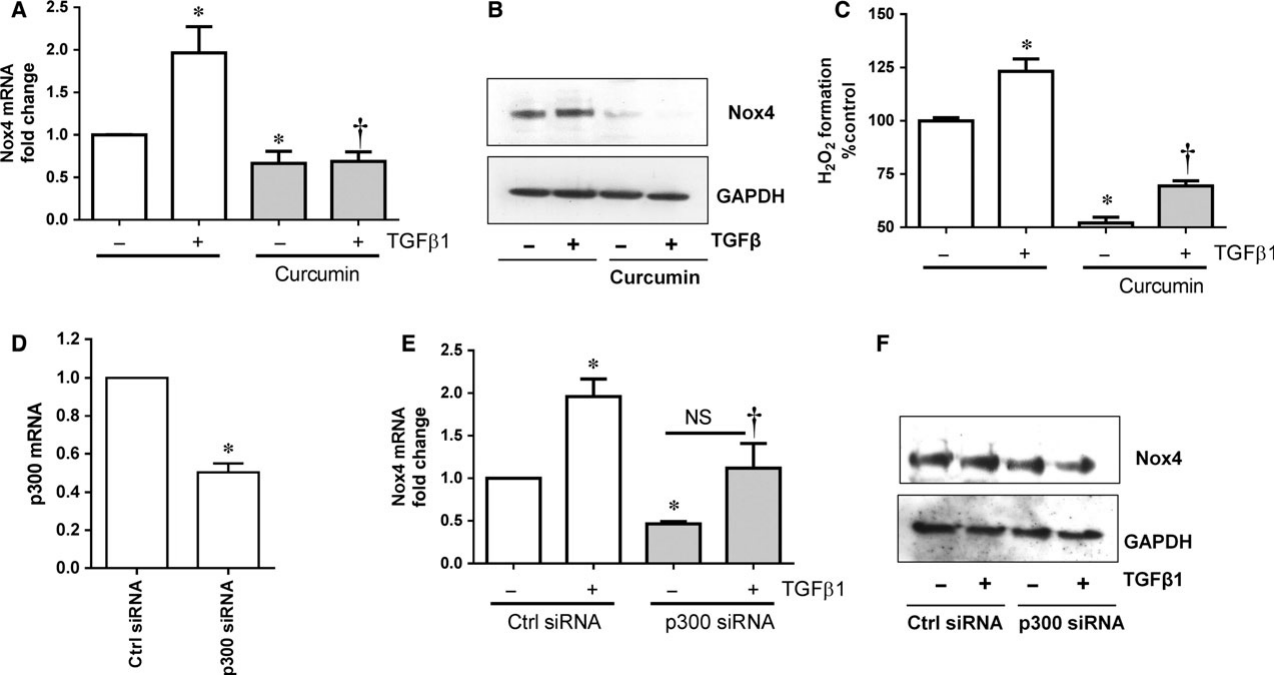
Effect of p300‐HAT inhibition on Nox4 expression and H_2_O_2_ formation in HMECs. (**A**) Pre‐treatment (1 hr) of HMECs with p300 inhibitor curcumin (50 μM) suppressed the basal and TGFβ1 (10 ng/ml‐6 hrs) induced on (**A**) Nox4 gene expression and (**B**) Nox4 protein expression as shown in a representative Western blot. (**C**) Basal and TGFβ1 (10 ng/ml‐6 hrs)‐induced H_2_O_2_ generation was reduced in HMECs pre‐treated with curcumin. (**D**) p300‐HAT siRNA markedly inhibits expression of p300 mRNA in HMECs. Down‐regulation of p300 with siRNA reduced basal and TGFβ1 (10 ng/ml‐6 hrs)‐induced (**E**) Nox4 gene expression and (**F**) Nox4 protein expression in HMECs. All data are mean ± S.E.M. from 4 to 6 independent experiments, *P < 0.05 from control without treatment; †*P* < 0.05 from treated cells with TGFβ1.

p300‐histone acetyltransferase is a co‐activator required for several transcription factors such as, Smad2/3 27, HIF‐1α 28 and cAMP response element‐binding protein (CREB) 7, 29. These transcription factors are also required for Nox4 gene expression 7, 30, 31. Previously, we have shown that inhibition of p300‐HAT expression or activity decreased Nox4 expression in endothelial cells 7. Similarly, curcumin a known inhibitor of p300‐HAT 32, also reduced TGFβ1‐induced Nox4 mRNA (Fig. 3A), protein (Fig. 3B) and H_2_O_2_ formation (Fig. 3C) in HMECs. In addition, we confirmed effects of p300‐HAT suppression using small interference RNA (siRNA; Fig. 3D). As expected, knockdown of p300‐HAT gene inhibits both basal and TGFβ1‐induced Nox4 mRNA (Fig. 3E) and protein (Fig. 3F) expression in HMECs suggesting p300‐HAT is essential for Nox4 expression.

**Figure 4:**
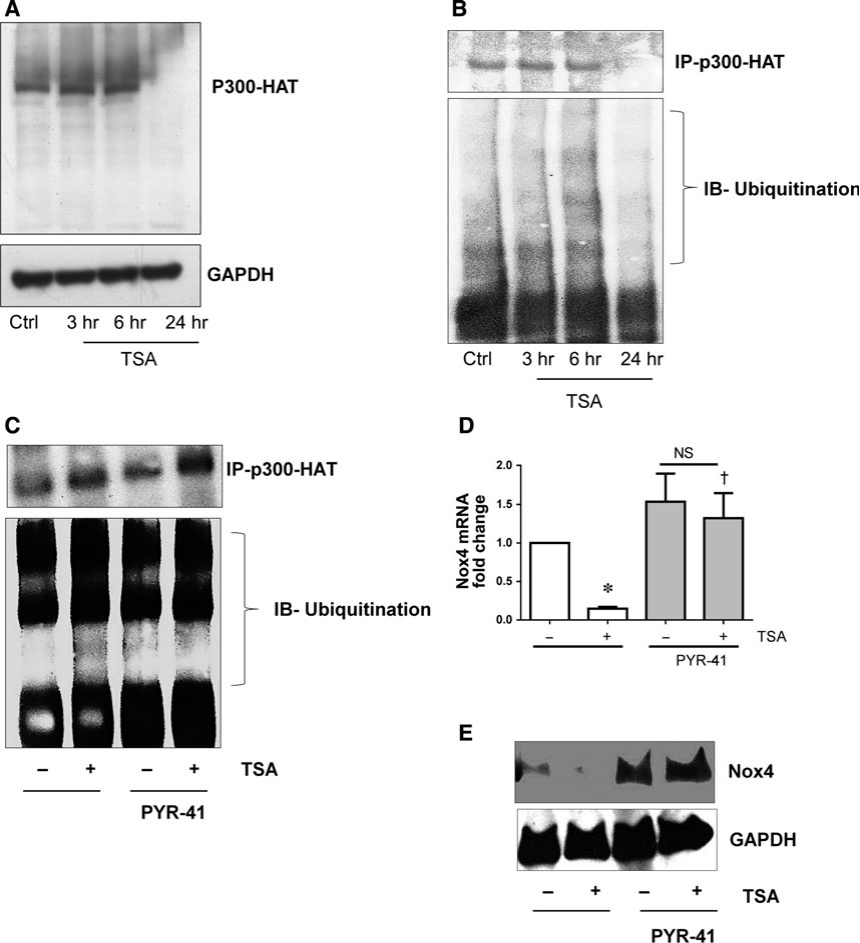
Effect of TSA induced p300‐HAT degradation *via* ubiquitin proteasome pathway and inhibits Nox4 expression in HMECs. (**A**) Trichostatin A (TSA; 0.33 μM) induced degradation of p300‐HAT protein at 24 hrs as shown in a representative Western blot. (**B**) TSA enhances ubiqutination of p300‐HAT at protein 3 and 6 hrs. TSA (0.33 μM‐6 hrs) treated HMECs cell lysates were immunoprecipitated with anti‐p300‐HAT antibody, immunoblotted and probed with anti‐ubiqutin antibody. (**C**) Inhibition of ubiquitin‐activating enzyme E1 using PYR41 (2.5 μM) block TSA (0.33 μM‐6 hrs) induced ubiqutination of p300‐HAT. (**D**) Inhibition of ubiquitin‐activating enzyme E1 using PYR41 (2.5 μM) reverted TSA (0.33 μM‐6 hrs) induced inhibition of Nox4 gene expression and (**E**) protein expression as shown in a representative Western blot. All data are mean ± S.E.M. from 3 to 4 independent experiments. *P < 0.05 from control without treatment; †*P* < 0.05 from treated cells with TSA.

Generally, HDAC inhibitors cause hyperacetylation of proteins by enhancing HATs activity. Therefore, it could be predicted that inhibition of HDAC ought to enhance HATs activity and Nox4 expression. Surprisingly we found that inhibition of both HDACs and p300‐HAT abrogated Nox4 expression in HMECs and HUVECs. To gain mechanistic insights, we evaluated the impact of TSA on p300‐HAT protein. Interestingly, p300‐HAT protein was not present after 24 hrs of TSA treatment in HMECs (Fig. 4A). Degradation of p300‐HAT could occur *via* ubiqutination‐proteasomes dependent mechanism 33. Therefore, we performed immunoprecipitation of p300‐HAT and investigated ubiqutination after TSA treatment. As shown in Figure 4B, p300‐HAT ubiqutination occurred as early as 3 hrs and reached maximum at 6 hrs followed by degradation after 24 hrs of TSA treatment. This indicates that TSA enhances early inactivation of p300‐HAT *via* ubiquitination leading proteasomal degradation. Inhibition of ubiquitination pathway using the ubiquitin‐activating enzyme E1 inhibitor PYR‐41 (2.5 μM) reduced TSA‐mediated ubiquitination of p300‐HAT (Fig. 4C). PYR‐41 also reversed TSA‐mediated abolition of Nox4 mRNA (Fig. 4D) and protein (Fig. 4E) in HMECs. Thus, TSA abrogated Nox4 expression *via* inactivation of p300‐HAT.

### Trichostatin A reduces endothelial cell migration *via* Nox4 signalling

**Figure 5:**
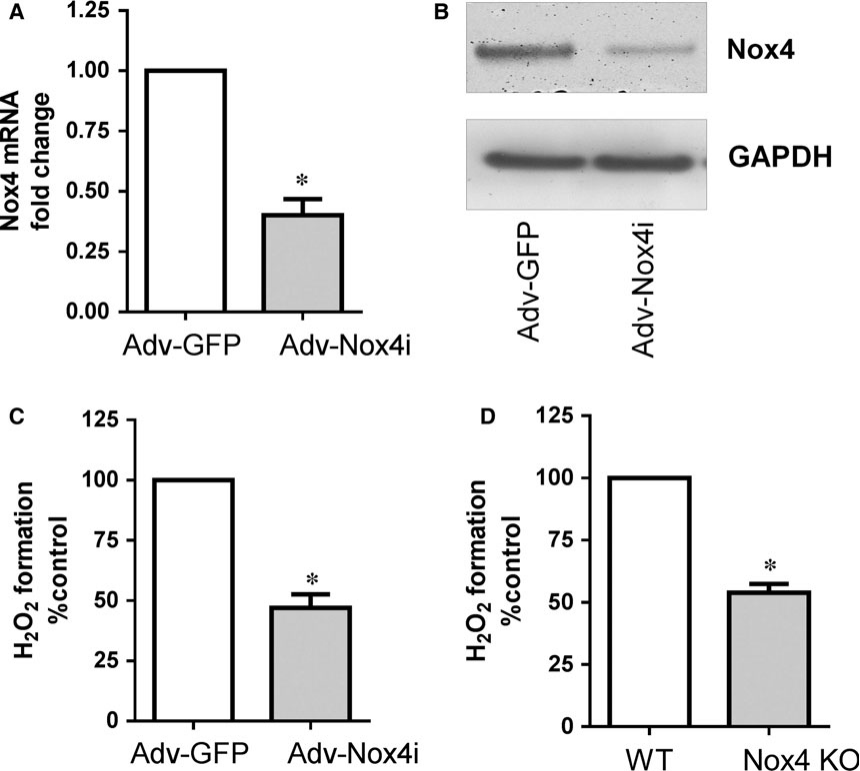
Effect of Nox4 inhibition on H_2_O_2_ production in endothelial cells. Treatment of HMECs with Adv‐Nox4i 72 hrs suppressed (**A**) Nox4 gene and (**B**) Nox4 protein expression, as shown in a representative Western blot and (**C**) H_2_O_2_ generation. (**D**) Similarly, wild type (WT) mouse lung endothelial cells (MLECs) derived H_2_O_2_ generation was less compared to Nox4 knockout (Nox4 KO) MLECs. All data are mean ± S.E.M. from 3 to 4 independent experiments, *P < 0.05 from Adv‐GFP or WT control.

Previously, we and others have shown that Nox4 enhances endothelial cell migration 5, 7, 34. We therefore attempted to clarify whether the effect of TSA on endothelial cell migration required Nox4 signalling. To illustrate the functional importance of HDAC inhibitor TSA *via* Nox4, first we used an adenovirus carrying RNA interference targeting human Nox4 (Adv‐Nox4i) to down regulate Nox4, which markedly reduced Nox4 mRNA expression (Fig. 5A), protein (Fig. 5B), and the formation of H_2_O_2_ (Fig. 5C) in HMECs. Similarly, the formation of H_2_O_2_ was lower in MLEC derived from Nox4 KO mice compared to the WT‐derived MLEC (Fig. 5D). Thus, Nox4 is a constitutively active source of H_2_O_2_ in endothelial cells and these were used further to evaluate cell migration and pro‐angiogenic activity *in vitro*.

**Figure 6:**
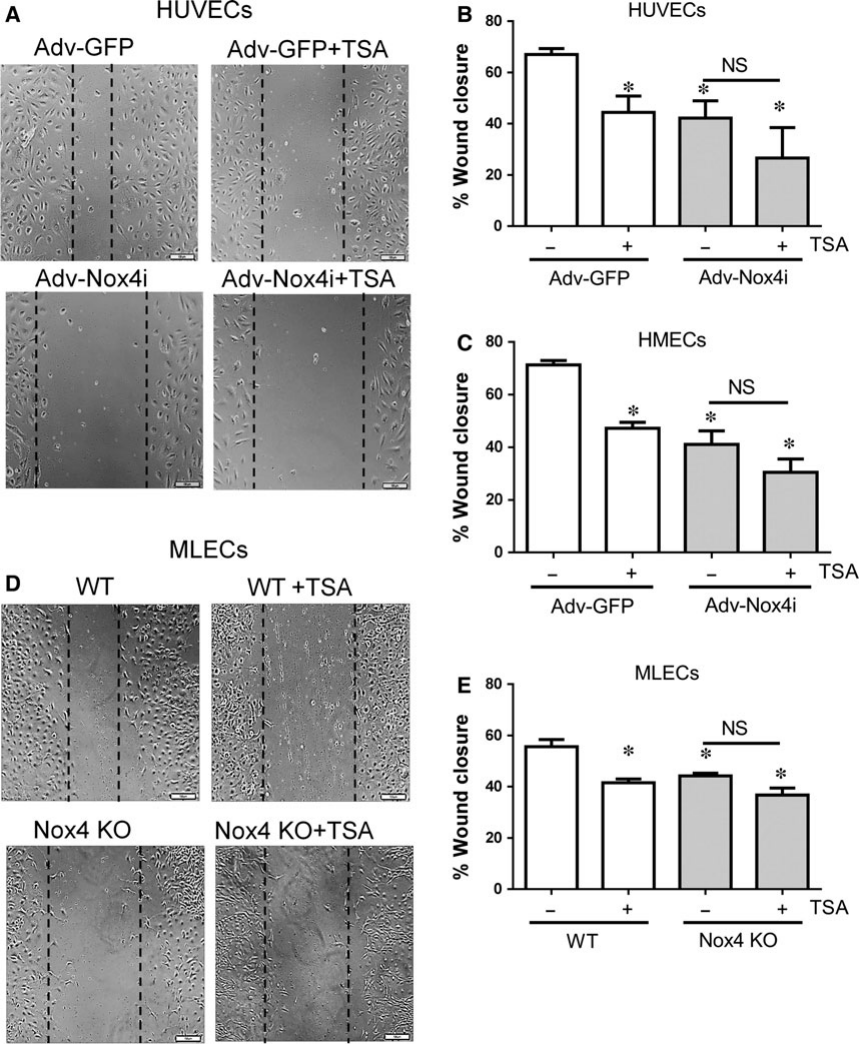
Effect of TSA on endothelial cell migration. HUVEC and HMECs were infected with either Adv‐GFP or Adv‐Nox4i for 48 hrs. EGM‐2 medium were used to induce endothelial cell migration in scratch assay. (**A**) Representative high magnification images of a single wound‐scratched healing assay performed on (**A**) HUVECs and (**D**) MLECs (scale bar represents 100 μm). (**B**) HUVECs and (**C**) HMECs cells were treated with trichostatin A (TSA; 0.33 μM) immediately after scratch for 16 hrs. (**E**) Similarly, wild type (WT) MLECs and Nox4 knockout (Nox4 KO) MLECs were also treated with TSA (0.33 μM) for 16 hrs. Cell migration was measured as percentage wound closer to time zero. Data expressed as mean ±S.E.M. from 4 to 6 independent experiments. *P < 0.05 from Adv‐GFP or WT without TSA treatment. NS: Not significant.

Disruption of the intact layer of endothelial cells using a scratch assay model causes migration of adjacent cells to fill in the wounded areas. Growth medium in control HUVEC and HMECs accelerates wound closure (Fig. 6A–C) which was inhibited by TSA and Adv‐Nox4i. Importantly, TSA did not further reduce wound closure in Nox4 depleted cells (Fig. 6A–C). Similarly, wound closure was slower in Nox4 KO MLECs and TSA treated WT MLECs compared to untreated WT MLECs (Fig. 6D and E). This effect of TSA was not observed in Nox4 KO MLECs (Fig. 6D and E). These data indicate that TSA does not have significant effects on endothelial cell migration in absence of Nox4 signalling.

### Trichostatin A suppresses tube formation *via* Nox4 signalling

**Figure 7:**
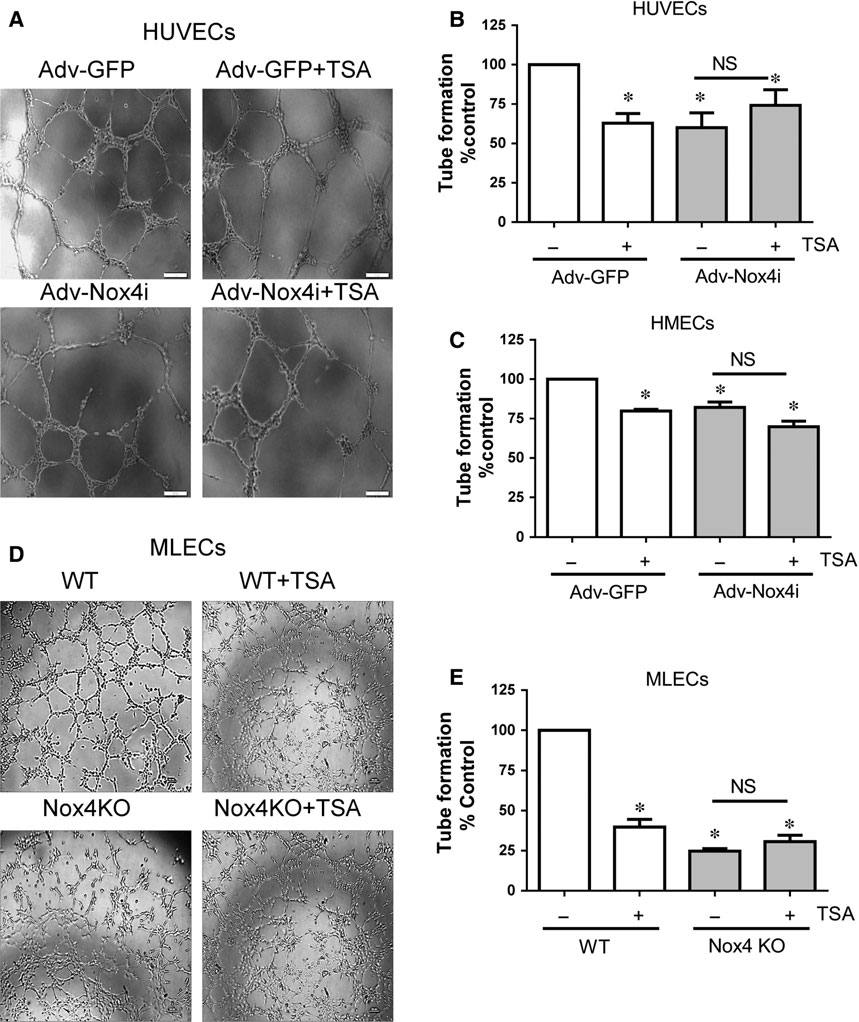
Effect of TSA on endothelial cell capillary formation. HUVECs and HMECs were infected with either Adv‐GFP or Adv‐Nox4i for 48 hrs. HUVECs HMECs and MLECs formed capillaries on growth factor reduced Matrigel^®^ at 8 hrs. (**A**) Representative high magnification images of a tube formation in (**A**) HUVECs and (**D**) MLECs (scale bar represents 20 μm). (**B**) HUVEC and (**C**) HMEC cells were treated at the cell seeding on Matrigel^®^ with tricostatin A (TSA; 0.33 μM) for 8 hrs. (**C**) Similarly, wild type (WT) MLECs and Nox4 knockout (Nox4 KO) MLECs were also treated with TSA (0.33 μM) for 8 hrs. Tube length was measured using ImageJ software and expressed as percentage control. Data expressed as mean ± S.E.M. from 4 to 6 independent experiments. *P < 0.05 from Adv‐GFP or WT without TSA treatment. NS: Not significant.

It is well‐known that TSA has anti‐angiogenic activity, however, involvement of Nox4 signalling in this process is not known yet. Endothelial cells involve cellular organization into a network of tube‐like structures. Capillary‐like tube formation was assessed by plating HUVECs and HMECs on solidified growth factor reduced Matrigel. Endothelial cells formed tube like structures within 8 hrs as shown in representative pictures of HUVECs (Fig. 7A) and WT MLECs (Fig. 7D). TSA and Adv‐Nox4i prevent capillary‐like structure formation to a similar extent in HUVECs and HMECs compared to their controls (Fig. 7A–C). This effect of TSA was not significantly different in Nox4 silenced HUVECs and HMECs (Fig. 7A–C). Similarly, tube formation was reduced in Nox4 KO MLECs and TSA‐treated WT MLECs compared to control WT MLECs (Fig. 7D and E). The effect of TSA was not observed in Nox4 KO MLECs (Fig. 7D and E). Thus, TSA exerts no significant effect on *in vitro* angiogenesis in the absence of Nox4 signalling.

### Trichostatin A reduces TGFβ1‐induced angiogenesis *in vivo*

**Figure 8:**
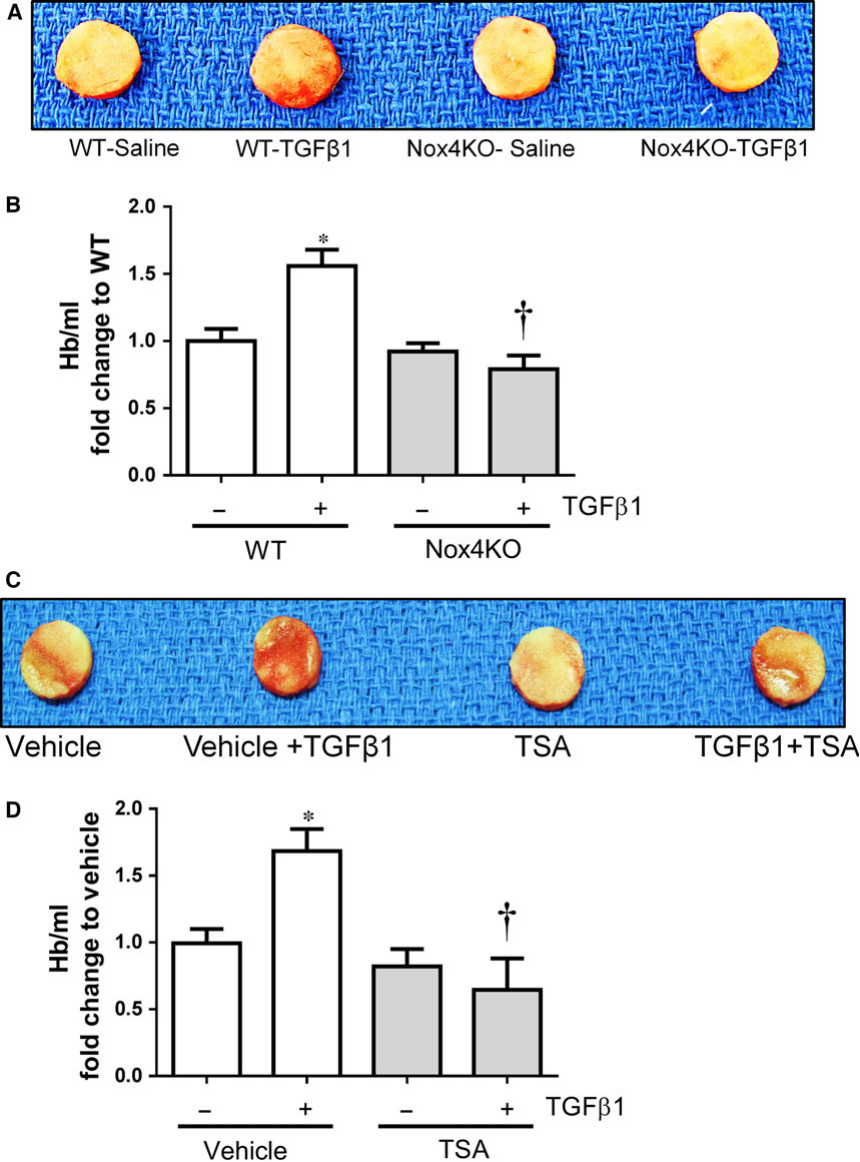
Effect of TSA on angiogenesis *in vivo*. (**A**) Representative picture of polyvinyl alcohol (PVA) sponges excised from wild type (WT) and Nox4 knockout (Nox4 KO) mice after 14 days. (**B**) Vascularization within sponges was determined quantitatively by haemoglobin content as an index of angiogenesis and expressed as % Hb/ml of WT (mean ± S.E.M. from *n* = 6). (**C**) Representative picture of PVA sponges excised from vehicle and TSA (1 mg/kg/48 hrs) mice after 14 days. (**D**) Vascularization within sponges was determined quantitatively by haemoglobin assay and expressed as % Hb/ml of vehicle (mean ± S.E.M. from *n* = 4). *P < 0.05 from wild type with saline or vehicle treatment; †*P* < 0.05 from wild type with TGFβ1 treatment.

Previously, we demonstrated that TGFβ1 induced angiogenesis in a mouse sponge model and this response to TGF‐β1 completely blocked in Nox4 KO (Exon 14‐15 deletion) mice 5. Here, we have used the same model but a different strain of Nox4 KO (Exon 4‐5 deletion) mice 26 kindly provided by Prof Karl‐Heinz Krause (University of Geneva). As in our previous study, we instilled the sponges with either saline or TGFβ1 (10 ng/ml) subcutaneously under the dorsal skin of WT littermates and Nox4 KO mice for 14 days to allow vessels to grow into the sponges. The degree of perfused vessel growth in the sponges was then evaluated by quantification of haemoglobin content in sponges. TGFβ1‐treated sponges harvested from WT mice showed greater vascularization (Fig. 8A) and higher haemoglobin content (Fig. 8B) compared to the saline‐treated sponges, suggesting TGFβ1‐induced vessels were perfused with blood. Most importantly, the augmenting effects of TGFβ1 on haemoglobin content were completely abolished in Nox4 KO mice (Fig. 8A and B). Given that TSA strongly inhibits Nox4 in endothelial cells and TGFβ1 induced angiogenesis *via* a Nox4‐dependent pathway *in vivo*, we investigated whether TSA could specifically inhibit TGFβ1‐induced angiogenesis *in vivo*. Indeed, the mice treated with TSA (1 mg/kg intraperitoneal) reduced substantially TGFβ1‐induced haemoglobin content in the sponges compared to the vehicle (1% DMSO in saline)‐treated mice (Fig. 8C and D). There was no difference observed in haemoglobin content in the saline instilled sponges (in absence of TGFβ1) derived from WT and TSA‐treated WT mice (Fig. 8C and D). These findings suggest that TSA inhibits TGFβ1‐induced angiogenesis *in vivo* and this effect of TSA is similar to that seen in Nox4 KO mice. Collectively, our data suggest that TSA reduces angiogenesis *in vitro* and *in vivo* and this involves suppression of Nox4 signalling.

## Discussion

Here, we demonstrate that the HDAC inhibitor TSA reduced Nox4 expression and activity in endothelial cells derived from different vascular beds. In addition, TSA impaired angiogenic activity both *in vitro* and *in vivo* under non‐hypoxic conditions. We show that TSA reduces angiogenesis by suppressing a Nox4‐dependent pathway, at least *in vitro*. Mechanistically, TSA inhibits Nox4 expression *via* the ubiquitin‐dependent degradation of the co‐activator p300‐HAT in endothelial cells. These results reveal a novel action of the HDAC inhibitor TSA to feed back control of p300‐HAT co‐activator activity and thereby affect Nox4 expression in endothelial cells.

Several transcription factors such as HIF‐1α 28, Smad2/3 30, Sp3 35, Oct‐1 36 and c‐Jun 37 are involved in regulation of Nox4 gene expression. All of these transcription factors require one of the common co‐activators p300‐HAT for gene expression activity 27, 31, 38, 39 and thus p300‐HAT might make a suitable target to modulate Nox4 expression therapeutically. Indeed, our recent study provides direct evidence that p300‐HAT activity is vital for CREB‐mediated Nox4 expression 7. Similarly, both the p300‐HAT inhibitor curcumin 32 and p300 siRNA blocked basal and TGFβ1‐induced Nox4 expression and activity. This is consistent with our previous finding that p300‐HAT activity is essential for Nox4 expression 7.

It came as a surprise that inhibition of both HDAC and p300‐HAT appeared to abrogate Nox4 expression in endothelial cells. In general, HDAC inhibitors cause hyperacetylation of histones and non‐histone proteins due to sustained activity of HAT, hence increased gene expression. However, continuous activity of HATs would clearly be detrimental in terms of gene expression and additional regulatory mechanisms are needed to control HAT activity. Indeed, it has become evident that HDAC inhibitors such as TSA and NaB increase p300 protein degradation at 20 hrs *via* an ubiqutination‐proteasomal pathway in HeLa cells 33, 40. These results are consistent with our findings that TSA induced ubiqutination of p300‐HAT as early as 3 and 6 hrs followed by degradation by 24 hrs (Fig. 4A and B). Moreover, the present study shows that inhibition of the ubiquitin‐activating enzyme E1 using PYR41 prevents TSA‐mediated suppression of Nox4 (Fig. 4D and E). It is likely that TSA‐induced early ubiqutination of p300‐HAT which may bind inefficiently to the transcription factors, and this in turn suppresses Nox4 expression. This view is partially supported by recent findings (Siuda *et al*.) showing inhibition of c‐Jun binding to the Nox4 promoter region leads to decreased Nox4 expression in the presence of the HDAC inhibitor scriptide 37. Alternatively, TSA has been shown to increase expression of the cyclin‐dependent kinase inhibitor‐1 p21WAF1 and decrease expression of p300‐HAT in breast cancer cells 41. Therefore, one can speculate that inactivation of co‐activator p300‐HAT could lead to inefficient binding of c‐Jun or other transcription factors to the Nox4 promoter and thereby affect Nox4 expression.

Histone deacetylase inhibitors affect the expression or activity of several mediators of angiogenesis such as HIF‐1α, VEGF and eNOS. For example, under hypoxic conditions, TSA up‐regulated von Hippel‐Lindau activity leading to suppression of HIF‐1α activity and VEGF expression 22. Under non‐hypoxic conditions, TSA destabilizes eNOS mRNA leading to reduction of both eNOS protein and nitric oxide availability, thereby compromising angiogenesis. Moreover, this effect of TSA was not reversed by addition of the potent angiogenic factor VEGF suggesting the anti‐angiogenic effect of TSA under non‐hypoxic conditions is not primarily attributed to VEGF suppression but that an eNOS‐mediated alternative mechanism may be involved 24. We and others suggested that Nox4‐derived H_2_O_2_ is required for eNOS expression and angiogenesis 5, 9, 12. Under non‐hypoxic conditions, we observed TSA down‐regulates Nox4 expression as early as 3 hrs and maximally at 6 hrs (Fig. 1A) whereas eNOS gene expression is down‐regulated later (24 hrs) in both HUVECs and HMECs (Fig. 1F). TSA‐mediated reduction of eNOS gene expression at a later time point accords with a previous report by Rossig *et al*. 24. Thus, TSA‐mediated inhibition of Nox4‐ derived H_2_O_2_ may lead to decreased expression of eNOS and regulated angiogenesis. In addition, it has been shown that HDAC inhibitors up‐regulate anti‐angigogenic genes such as thrombospondin‐1 42 and the VEGF competitor semaphorin III 43. Hence, whether Nox4 signalling is required for up‐regulation of anti‐angiogenic genes by HDAC inhibitors warrants further investigation.

Histone deacetylase inhibitors have previously been shown to reduce proliferation, migration and angiogenesis *in vitro* 22, 23, 24, 44. These effects of HDAC inhibitors are therefore similar to those of Nox4 deficiency 5, 9, 25. We also demonstrated a minimal effect of TSA on migration and angiogenesis in a Nox4‐deficient cells suggesting Nox4‐depedent redox component is essential to switch off the angiogenic response. HDAC inhibitors also reduced Nox1 and Nox2 expressions in vascular smooth muscle cells and phagocytic cells, respectively 45, 46 but, we could not find such an effect in endothelial cells (Fig. 1C). Clearly the effect of HDAC inhibitors on expression of these Nox isoforms is dependent upon cell type. In addition, we show increased Nox5 expression after 24 hrs TSA treatment (Fig. 1C), but H_2_O_2_ production remained low, suggesting only Nox4 is involved in TSA‐mediated anti‐angiogenic effects in endothelial cells.

The physiological and pathophysiological roles of Nox4 remain to be fully elucidated. However, increased Nox4 expression or activity has been observed during hypoxia or TGFβ1‐induced angiogenesis as well as a number of pathological conditions including haemangioma (endothelial tumour cells) formation 47, retinal neovascularization 48, 49, cardiac failure 50, fibrosis 51, pulmonary hypertension 52 and stroke 53. The effects of TSA‐mediated inhibition of Nox4 expression is not limited to endothelial cells but is also observed in human dermal fibroblasts (Fig. 2E) suggesting that HDAC inhibitors may have similar effects on Nox4 expression in different cell types. Hence, the inhibition of Nox4‐depedent redox signalling using HDAC inhibitors may be an interesting approach to investigate several pathological conditions given the success of HDAC inhibitors in the clinic for cancer ablation 54.

**Figure 9:**
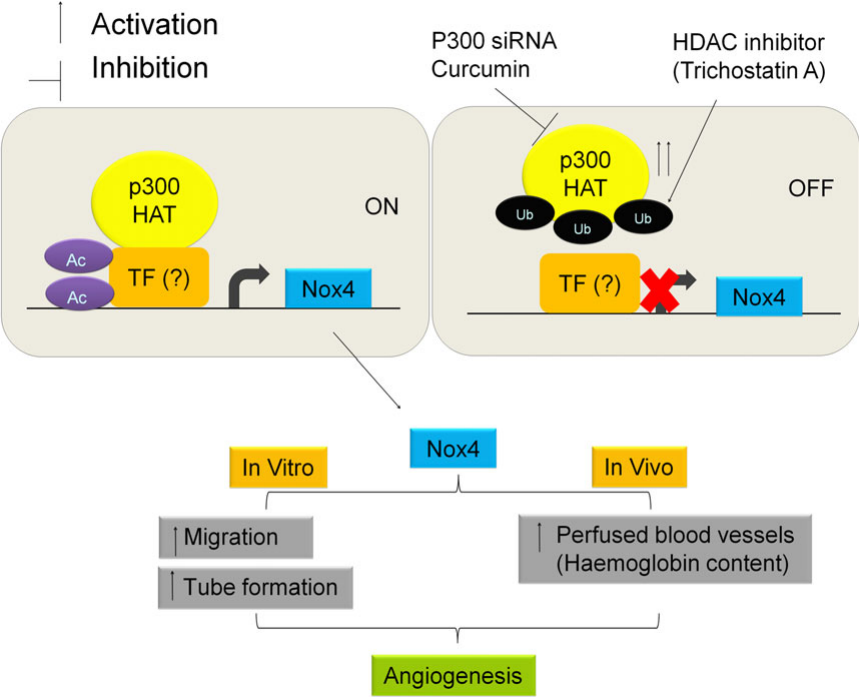
Schematic for HDAC inhibitor trichostatin A reduced Nox4 and angiogenesis *via* inhibition of p300‐HAT. P300‐HAT activity such as acetylation (Ac) of transcription factor (TF) required for Nox4 expression in endothelial cells. Trichostatin A enhanced ubiquitination (Ub) of p300‐HAT and reduced expression of Nox4 as well as angiogenic activity in *in vitro* and *in vivo*.

In conclusion, the interaction between p300‐HAT and HDACs plays an important role in the regulation of Nox4 expression in endothelial cells. Mechanistically, it is likely that HDAC inhibitor TSA reduce expression of Nox4 *via* ubiquitin‐mediated inactivation of p300‐HAT. Nox4 plays vital roles in endothelial cell functions such as migration and angiogenesis (Fig. 9). *In vitro* anti‐angiogenic effects of TSA under non‐hypoxic conditions appear to be exerted *via* a Nox4‐dependent pathway. TSA also inhibits TGFβ1‐induced angiogenesis *in vivo* and this effect of TSA is similar to that of Nox4 ablation. Clearly HDAC inhibitors regulate angiogenesis *in vitro* and *in vivo via* a Nox4‐dependent pathway.

## Conflicts of interest

The authors confirm that there are no conflicts of interest.

## Author contribution

N.Y.H. performed experiments, data interpretation and writing of the manuscript, H.M.P. study design, performed experiments, data interpretation and writing of the manuscript. G. J.D. funded all experiments and edited the manuscript.

## References

[1] Drummond GR , Selemidis S , Griendling KK , *et al* Combating oxidative stress in vascular disease: NADPH oxidases as therapeutic targets. Nat Rev Drug Discov. 2011; 10: 453–71. 21629295 10.1038/nrd3403 PMC3361719

[2] Bedard K , Krause KH . The NOX family of ROS‐generating NADPH oxidases: physiology and pathophysiology. Physiol Rev. 2007; 87: 245–313. 17237347 10.1152/physrev.00044.2005

[3] Chan EC , Jiang F , Peshavariya HM , *et al* Regulation of cell proliferation by NADPH oxidase‐mediated signaling: potential roles in tissue repair, regenerative medicine and tissue engineering. Pharmacol Ther. 2009; 122: 97–108. 19285105 10.1016/j.pharmthera.2009.02.005

[4] Takac I , Schroder K , Zhang L , *et al* The E‐loop is involved in hydrogen peroxide formation by the NADPH oxidase Nox4. J Biol Chem. 2011; 286: 13304–13. 21343298 10.1074/jbc.M110.192138 PMC3075677

[5] Peshavariya HM , Chan EC , Liu GS , *et al* Transforming growth factor‐beta1 requires NADPH oxidase 4 for angiogenesis *in vitro* and *in vivo* . J Cell Mol Med. 2014; 18: 1172–83. 24629065 10.1111/jcmm.12263 PMC4508156

[6] Hu T , RamachandraRao SP , Siva S , *et al* Reactive oxygen species production *via* NADPH oxidase mediates TGF‐β‐induced cytoskeletal alterations in endothelial cells. Am J Physiol Renal Physiol. 2005; 289: F816–25. 16159901 10.1152/ajprenal.00024.2005 PMC1460011

[7] Peshavariya HM , Liu GS , Chang CW , *et al* Prostacyclin signaling boosts NADPH oxidase 4 in the endothelium promoting cytoprotection and angiogenesis. Antioxid Redox Signal. 2014; 20: 2710–25. 24450852 10.1089/ars.2013.5374

[8] Basuroy S , Bhattacharya S , Leffler CW , *et al* Nox4 NADPH oxidase mediates oxidative stress and apoptosis caused by TNF‐α in cerebral vascular endothelial cells. Am J Physiol Cell Physiol. 2009; 296: C422–32. 19118162 10.1152/ajpcell.00381.2008 PMC2660262

[9] Craige SM , Chen K , Pei Y , *et al* NADPH oxidase 4 promotes endothelial angiogenesis through endothelial nitric oxide synthase activation. Circulation. 2011; 124: 731–40. 21788590 10.1161/CIRCULATIONAHA.111.030775 PMC3589548

[10] Wang J , Hong Z , Zeng C , *et al* NADPH oxidase 4 promotes cardiac microvascular angiogenesis after hypoxia/reoxygenation *in vitro* . Free Radic Biol Med. 2014; 69: 278–88. 24480752 10.1016/j.freeradbiomed.2014.01.027

[11] Peshavariya H , Dusting GJ , Jiang F , *et al* NADPH oxidase isoform selective regulation of endothelial cell proliferation and survival. Naunyn‐Schmiedeberg's Arch Pharmacol. 2009; 380: 193–204. 19337723 10.1007/s00210-009-0413-0

[12] Chen L , Xiao J , Kuroda J , *et al* Both hydrogen peroxide and transforming growth factor beta 1 contribute to endothelial Nox4 mediated angiogenesis in endothelial Nox4 transgenic mouse lines. Biochim Biophys Acta. 2014; 1842: 2489–99. 25315297 10.1016/j.bbadis.2014.10.007

[13] Helfinger V , Henke N , Harenkamp S , *et al* The NADPH Oxidase Nox4 mediates tumour angiogenesis. Acta Physiol. 2015; 216(4): 435–46. 10.1111/apha.12625 26513738

[14] Zhang M , Brewer AC , Schroder K , *et al* NADPH oxidase‐4 mediates protection against chronic load‐induced stress in mouse hearts by enhancing angiogenesis. Proc Natl Acad Sci USA. 2010; 107: 18121–6. 20921387 10.1073/pnas.1009700107 PMC2964252

[15] Dekker FJ , Haisma HJ . Histone acetyl transferases as emerging drug targets. Drug Discovery Today. 2009; 14: 942–8. 19577000 10.1016/j.drudis.2009.06.008

[16] Wang F , Marshall CB , Ikura M . Transcriptional/epigenetic regulator CBP/p300 in tumorigenesis: structural and functional versatility in target recognition. Cell Mol Life Sci. 2013; 70: 3989–4008. 23307074 10.1007/s00018-012-1254-4 PMC11113169

[17] Chen W , Bacanamwo M , Harrison DG . Activation of p300 histone acetyltransferase activity is an early endothelial response to laminar shear stress and is essential for stimulation of endothelial nitric‐oxide synthase mRNA transcription. J Biol Chem. 2008; 283: 16293–8. 18397880 10.1074/jbc.M801803200 PMC2423243

[18] Bots M , Johnstone RW . Rational combinations using HDAC inhibitors. Clin Cancer Res. 2009; 15: 3970–7. 19509171 10.1158/1078-0432.CCR-08-2786

[19] Abend A , Kehat I . Histone deacetylases as therapeutic targets–from cancer to cardiac disease. Pharmacol Ther. 2015; 147: 55–62. 25444758 10.1016/j.pharmthera.2014.11.003

[20] Micelli C , Rastelli G . Histone deacetylases: structural determinants of inhibitor selectivity. Drug Discovery Today. 2015; 20: 718–35. 25687212 10.1016/j.drudis.2015.01.007

[21] Shakespear MR , Halili MA , Irvine KM , *et al* Histone deacetylases as regulators of inflammation and immunity. Trends Immunol. 2011; 32: 335–43. 21570914 10.1016/j.it.2011.04.001

[22] Kim MS , Kwon HJ , Lee YM , *et al* Histone deacetylases induce angiogenesis by negative regulation of tumor suppressor genes. Nat Med. 2001; 7: 437–43. 11283670 10.1038/86507

[23] Kim JH , Kim JH , Oh M , *et al* *N*‐hydroxy‐7‐(2‐naphthylthio) heptanomide inhibits retinal and choroidal angiogenesis. Mol Pharm. 2009; 6: 513–9. 19718802 10.1021/mp800178b

[24] Rossig L , Li H , Fisslthaler B , *et al* Inhibitors of histone deacetylation downregulate the expression of endothelial nitric oxide synthase and compromise endothelial cell function in vasorelaxation and angiogenesis. Circ Res. 2002; 91: 837–44. 12411399 10.1161/01.res.0000037983.07158.b1

[25] Schroder K , Zhang M , Benkhoff S , *et al* Nox4 is a protective reactive oxygen species generating vascular NADPH oxidase. Circ Res. 2012; 110: 1217–25. 22456182 10.1161/CIRCRESAHA.112.267054

[26] Carnesecchi S , Deffert C , Donati Y , *et al* A key role for NOX4 in epithelial cell death during development of lung fibrosis. Antioxid Redox Signal. 2011; 15: 607–19. 21391892 10.1089/ars.2010.3829 PMC3163392

[27] Nishihara A , Hanai JI , Okamoto N , *et al* Role of p300, a transcriptional coactivator, in signalling of TGF‐beta. Genes Cells. 1998; 3: 613–23. 9813111 10.1046/j.1365-2443.1998.00217.x

[28] Diebold I , Petry A , Hess J , *et al* The NADPH oxidase subunit NOX4 is a new target gene of the hypoxia‐inducible factor‐1. Mol Biol Cell. 2010; 21: 2087–96. 20427574 10.1091/mbc.E09-12-1003 PMC2883952

[29] Yuan LW , Gambee JE . Histone acetylation by p300 is involved in CREB‐mediated transcription on chromatin. Biochim Biophys Acta. 2001; 1541: 161–9. 11755210 10.1016/s0167-4889(01)00141-0

[30] Bai G , Hock TD , Logsdon N , *et al* A far‐upstream AP‐1/Smad binding box regulates human NOX4 promoter activation by transforming growth factor‐beta. Gene. 2014; 540: 62–7. 24560583 10.1016/j.gene.2014.02.026 PMC4009368

[31] Gu J , Milligan J , Huang LE . Molecular mechanism of hypoxia‐inducible factor 1alpha ‐p300 interaction. A leucine‐rich interface regulated by a single cysteine. J Biol Chem. 2001; 276: 3550–4. 11063749 10.1074/jbc.M009522200

[32] Marcu MG , Jung YJ , Lee S , *et al* Curcumin is an inhibitor of p300 histone acetylatransferase. Med Chem. 2006; 2: 169–74. 16787365 10.2174/157340606776056133

[33] Li Q , Su A , Chen J , *et al* Attenuation of glucocorticoid signaling through targeted degradation of p300 *via* the 26S proteasome pathway. Mol Endocrinol. 2002; 16: 2819–27. 12456802 10.1210/me.2002-0154

[34] Pendyala S , Gorshkova IA , Usatyuk PV , *et al* Role of Nox4 and Nox2 in hyperoxia‐induced reactive oxygen species generation and migration of human lung endothelial cells. Antioxid Redox Signal. 2009; 11: 747–64. 18783311 10.1089/ars.2008.2203 PMC2850303

[35] Katsuyama M , Hirai H , Iwata K , *et al* Sp3 transcription factor is crucial for transcriptional activation of the human NOX4 gene. FEBS J. 2011; 278: 964–72. 21235713 10.1111/j.1742-4658.2011.08018.x

[36] Goettsch C , Goettsch W , Brux M , *et al* Arterial flow reduces oxidative stress *via* an antioxidant response element and Oct‐1 binding site within the NADPH oxidase 4 promoter in endothelial cells. Basic Res Cardiol. 2011; 106: 551–61. 21399967 10.1007/s00395-011-0170-3

[37] Siuda D , Zechner U , El Hajj N , *et al* Transcriptional regulation of Nox4 by histone deacetylases in human endothelial cells. Basic Res Cardiol. 2012; 107: 1–12. 10.1007/s00395-012-0283-3 22791246

[38] Chan HM , La Thangue NB . p300/CBP proteins: HATs for transcriptional bridges and scaffolds. J Cell Sci. 2001; 114: 2363–73. 11559745 10.1242/jcs.114.13.2363

[39] Ammanamanchi S , Freeman JW , Brattain MG . Acetylated sp3 is a transcriptional activator. J Biol Chem. 2003; 278: 35775–80. 12837748 10.1074/jbc.M305961200

[40] Chen J , St‐Germain JR , Li Q . B56 regulatory subunit of protein phosphatase 2A mediates valproic acid‐induced p300 degradation. Mol Cell Biol. 2005; 25: 525–32. 15632055 10.1128/MCB.25.2.525-532.2005 PMC543421

[41] Tan HH , Porter AG . p21(WAF1) negatively regulates DNMT1 expression in mammalian cells. Biochem Biophys Res Commun. 2009; 382: 171–6. 19275888 10.1016/j.bbrc.2009.03.001

[42] Kang JH , Kim SA , Chang SY , *et al* Inhibition of trichostatin A‐induced antiangiogenesis by small‐interfering RNA for thrombospondin‐1. Exp Mol Med. 2007; 39: 402–11. 17603295 10.1038/emm.2007.45

[43] Deroanne CF , Bonjean K , Servotte S , *et al* Histone deacetylases inhibitors as anti‐angiogenic agents altering vascular endothelial growth factor signaling. Oncogene. 2002; 21: 427–36. 11821955 10.1038/sj.onc.1205108

[44] Michaelis M , Michaelis UR , Fleming I , *et al* Valproic acid inhibits angiogenesis *in vitro* and *in vivo* . Mol Pharmacol. 2004; 65: 520–7. 14978230 10.1124/mol.65.3.520

[45] Siuda D , Tobias S , Rus A , *et al* Dexamethasone upregulates Nox1 expression in vascular smooth muscle cells. Pharmacology. 2014; 94: 13–20. 25171190 10.1159/000365932

[46] Mombelli M , Lugrin J , Rubino I , *et al* Histone deacetylase inhibitors impair antibacterial defenses of macrophages. J Infect Dis. 2011; 204: 1367–74. 21921209 10.1093/infdis/jir553

[47] Bhandarkar SS , Jaconi M , Fried LE , *et al* Fulvene‐5 potently inhibits NADPH oxidase 4 and blocks the growth of endothelial tumors in mice. J Clin Invest. 2009; 119: 2359–65. 19620773 10.1172/JCI33877 PMC2719922

[48] Wang H , Yang Z , Jiang Y , *et al* Endothelial NADPH oxidase 4 mediates vascular endothelial growth factor receptor 2‐induced intravitreal neovascularization in a rat model of retinopathy of prematurity. Mol Vision. 2014; 20: 231–41. PMC3945806 24623966

[49] Li J , Wang JJ , Zhang SX . NADPH oxidase 4‐derived H_2_O_2_ promotes aberrant retinal neovascularization *via* activation of VEGF receptor 2 pathway in oxygen‐induced retinopathy. J Diabetes Res. 2015; 2015: 963289. 25866826 10.1155/2015/963289 PMC4381975

[50] Kuroda J , Ago T , Matsushima S , *et al* NADPH oxidase 4 (Nox4) is a major source of oxidative stress in the failing heart. Proc Natl Acad Sci USA. 2010; 107: 15565–70. 20713697 10.1073/pnas.1002178107 PMC2932625

[51] Hecker L , Vittal R , Jones T , *et al* NADPH oxidase‐4 mediates myofibroblast activation and fibrogenic responses to lung injury. Nat Med. 2009; 15: 1077–81. 19701206 10.1038/nm.2005 PMC2743335

[52] Barman SA , Chen F , Su Y , *et al* NADPH oxidase 4 is expressed in pulmonary artery adventitia and contributes to hypertensive vascular remodeling. Arterioscler Thromb Vasc Biol. 2014; 34: 1704–15. 24947524 10.1161/ATVBAHA.114.303848 PMC4228789

[53] Kleinschnitz C , Grund H , Wingler K , *et al* Post‐stroke inhibition of induced NADPH oxidase type 4 prevents oxidative stress and neurodegeneration. PLoS Biol. 2010; 8(9): e1000479. 20877715 10.1371/journal.pbio.1000479 PMC2943442

[54] Falkenberg KJ , Johnstone RW . Histone deacetylases and their inhibitors in cancer, neurological diseases and immune disorders. Nat Rev Drug Discov. 2014; 13: 673–91. 25131830 10.1038/nrd4360

